# Statin use and the risk of tubulointerstitial nephritis: a real-world signal detection analysis using FDA Adverse Event Reporting System

**DOI:** 10.3389/fmed.2025.1674331

**Published:** 2025-11-11

**Authors:** Ayesha Yasmeen, Mamoon H. Syed, Amani Khardali, Hadi A. Almansour, Thamir M. Alshammari

**Affiliations:** 1 Department of Clinical Practice, College of Pharmacy, Jazan University, Jazan, Saudi Arabia; 2 Pharmacy Practice Research Unit, College of Pharmacy, Jazan University, Jazan, Saudi Arabia

**Keywords:** hydroxymethylglutaryl-CoA reductase inhibitors, tubulointerstitial nephritis, pharmacovigilance, adverse drug reaction reporting systems, drug surveillance, postmarketing

## Abstract

**Background:**

Statins are widely prescribed for cardiovascular risk reduction but have been linked to a range of adverse effects. Tubulointerstitial nephritis (TIN), a potentially serious renal condition, has been sporadically reported with statin use. This study aims to evaluate the association between statins and TIN using real-world pharmacovigilance data.

**Methods:**

We analyzed adverse event reports from the U.S. FDA Adverse Event Reporting System (FAERS) between 2017 and 2024. Cases of TIN associated with statins were identified using MedDRA Preferred Terms. Disproportionality analyses—including Reporting Odds Ratio (ROR), Proportional Reporting Ratio (PRR), Empirical Bayes Geometric Mean (EBGM), and Information Component (IC)—were applied to detect safety signals, stratified by pre- and post-COVID-19 periods.

**Results:**

A total of 120 TIN cases linked to statin use were identified. While no significant signal was detected prior to 2020, consistent signal emergence was noted from 2021 onwards. In 2024, all signal metrics peaked (e.g., ROR 5.77; PRR 5.75; EBGM 5.32; IC 2.41), meeting thresholds for signal detection. Most reports involved patients aged ≥ 60 years and over 65% resulted in hospitalization. Geographic analysis showed wide international distribution, with the majority of reports from the United States and Europe.

**Conclusion:**

This real-world analysis identifies a statistically significant disproportionality signal that indicates a possible association between statin use and TIN, particularly in older adults. Clinicians may consider TIN in patients presenting with unexplained renal dysfunction while on statins. Further research is warranted to evaluate this association and identify patient-level risk factors.

## Introduction

The American Heart Association reports cardiovascular disease (CVD) as the leading cause of mortality in the United States (US) (1). The efficacy of statins in reducing CVD mortality and morbidity is well-established, and their cholesterol-lowering properties play a significant role in this reduction (2). In individuals aged between 40 and 75 years with one or more risk factors, such as dyslipidemia, hypertension, diabetes, or smoking, statins are recommended for the primary prevention of CVD (3). Statins are competitive inhibitors of Hydroxymethylglutaryl-CoA (HMG-CoA) reductase, an essential enzyme that controls cholesterol production in the liver. By inhibiting this enzyme, liver cholesterol synthesis decreases, and subsequently hepatocytes increase LDL receptor production, enhancing LDL cholesterol uptake and recycling, which reduces blood LDL cholesterol levels, thereby lowering cardiovascular risk (4).

Statins, which are generally well-tolerated and effective in reducing cardiovascular events, can cause various adverse events in some patients (5). The most common and significant side effects are neuromuscular, including myalgia, myopathy, and rhabdomyolysis in rare cases (6, 7). These muscle-related adverse events account for approximately two-thirds of all reported side effects associated with statin use (6, 8). Other potential adverse events include hepatotoxicity, new-onset diabetes, and cognitive impairment, although these are less common, and their association with statin use remains controversial (9).

Adverse effects of statins have also been reported in the FDA Adverse Event Reporting System (FAERS) (10). The FAERS database plays a crucial role in drug safety monitoring and regulatory decision-making (11). Studies analyzing FAERS data have revealed significant underreporting of adverse drug events (ADEs) for statins, with reporting rates ranging from 0.01 to 44% of expected events (10). Among patients treated with statins, especially simvastatin, rhabdomyolysis is a frequently reported adverse event (12). Tubulointerstitial nephritis (TIN) is another adverse event secondary to statin use, which has been reported in many case reports since 2013 (13–16). TIN is characterized by the presence of inflammatory cells such as lymphocytes, macrophages, and plasma cells infiltrating the kidney interstitium, often accompanied by tubulitis (17). Patients typically exhibit symptoms, such as rash, fever, eosinophilia, and increased levels of immunoglobulin E (IgE). However, the symptoms are frequently nonspecific, which can delay the diagnosis and treatment of the condition, potentially leading to kidney dysfunction (17). TIN is categorized as acute or chronic, based on cause, duration, and histological characteristics with drug-induced acute interstitial nephritis being the leading cause of TIN, accounting for 70–75% of cases (18).

A recent study utilizing data from the Japanese Adverse Drug Event Report database identified the drugs that most commonly induce TIN, including atorvastatin and rosuvastatin (17). Moreover, a recent report by the European Medicines Agency (EMA) outlined the recommendations adopted by the Pharmacovigilance Risk Assessment Committee (PRAC) on safety signals and requested supplementary information regarding the potential signal of tubulointerstitial nephritis associated with rosuvastatin use from the concerned Marketing Authorization Holder (MAH) (19). Considering these recent reports, our study aimed to identify and quantify potential safety signals related to TIN in statin users through a comprehensive analysis of the FAERS database. The findings from this real-world data study will provide valuable insights into the renal safety profile of statins and inform clinical decision-making regarding their use in patients at risk of kidney complications.

## Methods

This research analyzed data from FAERS database, covering reports submitted between 2017 and 2024. The analysis window (2017–2024) was selected a priori to reflect a recent period with consistent FAERS extract structure and contemporary MedDRA coding, and to enable a prespecified comparison of pre-pandemic (2017–2019) vs. pandemic/post-pandemic (2020–2024) reporting. Earlier FAERS years were not included to reduce heterogeneity from changes in reporting practices, indications, and labeling. The 2024 endpoint reflects the latest full year available at the time of extraction. FAERS compiles adverse drug reactions and medication errors associated with pharmaceuticals and biologics reported by patients, healthcare providers, manufacturers and legal professionals. FAERS database, publicly available at https://open.fda.gov/data/faers/, is updated every quarter a year (20). While FAERS is U.S.-based, it includes reports from across the globe, making it a valuable resource for pharmacovigilance studies and post-market surveillance research. All data are anonymized to protect individual privacy.

FAERS comprises seven file categories: Drug Information (DRUG), Adverse Events (REAC), Outcomes (OUTC), Demographic and Administrative Information (DEMO), Drug Therapy Dates (THER), Indications for Use (INDI) and Report Sources (RPSR). Reports are classified by the reporter's type including consumer, lawyers, healthcare professionals (e.g., physician, nurse or pharmacist), and the severity of outcomes (e.g., death, hospitalization, congenital anomalies). The reporter's country indicates the source of the latest submission, which may be domestic (i.e., US) or international. At the time of the adverse event, patient age is documented numerically (20, 21).

A unique identifier, PRIMARYID, linked all datasets. Pharmaceutical manufacturers are required by the US FDA to report adverse drug reactions (ADRs) for their products, while healthcare providers and patients are advised to submit reports voluntarily from all countries across the globe (22–24).

The study extracted FAERS data from 2017 to 2024 to identify and quantify potential safety signals related to TIN in statin users. Adverse event was coded using the Medical Dictionary for Regulatory Activities (MedDRA), with Preferred Terms (PTs) of “tubulointerstitial nephritis” used to identify relevant cases. The research team ensured and verified outputs to guarantee accuracy of relevant cases. The exposure of interest includes statin medications, including rosuvastatin, atorvastatin, simvastatin, pravastatin, pitavastatin, and lovastatin. This study followed the READUS-PV (Real-world Evidence from ADR Databases Used for Signal detection in Pharmacovigilance) guidelines for conducting FAERS-based pharmacovigilance research, as outlined in recently published best practice recommendations (25). These principles informed our approach to case identification, deduplication, and signal detection to enhance methodological rigor and reproducibility.

The analysis focused on potential safety indications related to TIN in statin users. Reports were filtered using DRUGNAME and PROD_AI variables, retaining only those where statin was the Primary Suspect (PS). Duplicates were removed using PRIMARYID, EVENT_DT, and PT variables.

The analysis focused on potential safety signals related to tubulointerstitial nephritis (TIN) in statin users. Reports were filtered using the FAERS DRUGNAME and PROD_AI fields, retaining only those in which a statin was coded as the Primary Suspect (PS). Duplicates were removed using PRIMARYID, EVENT_DT, and PT; where available, CASEVERSION was used to retain the most recent case version. Analyses were pre-specified at the statin-class level because molecule-specific comparisons in FAERS are prone to misinterpretation—exposure denominators/market share are unavailable, molecule-level strata are small (unstable), and stimulated/notoriety reporting and clinical channeling can differ across products; therefore per-molecule disproportionality was not planned or presented.

A case/non-case disproportionality analysis was conducted to evaluate statin's risks related to TIN in statin users. Cases were defined as TIN event reports linked to statin, while non-cases included all other adverse event reports for the drug. Both Bayesian and frequentist statistical methods were applied, using a 2 × 2 contingency table with four components: “a” (cases for the studied medications), “b” (non-cases for the studied medications), “c” (cases for other medications), and “d” (non-cases for other medications) (Table 1).

**Table 1:** Contingency table for disproportionality analyses.

	**Adverse drug reaction of interest**	**Other adverse drug reactions**
Drug of interest	a	b
All other drugs in the database	c	d

Key disproportionality metrics—Reporting Odds Ratio (ROR), Proportional Reporting Ratio (PRR), Empirical Bayes Geometric Mean (EBGM), and Information Component (IC)—were calculated. A safety signal was flagged if ROR > 1, PRR ≥ 2, EBGM > 2, or IC > 0 (23, 24). To ensure reliability, important signals were required to meet all four criteria. Analyses were accomplished using R software (v4.2.2) and R Studio (2024.04.2+764).

## Results

A total of 120 cases of tubulointerstitial nephritis (TIN) associated with statin use were identified in the FAERS database between 2017 and 2024. We stratified the analysis into two periods: before the COVID-19 pandemic (2017–2019) and during and after the COVID-19 pandemic (2020–2024) to assess the trends over time.

### Demographic and reporter characteristics

**Figure 1:**
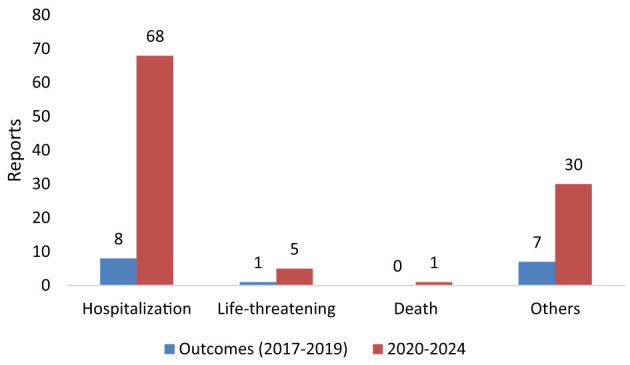
Outcomes of TIN events related to statin use.

Table 2 indicates the reports of TIN cases associated with statin use in the period before the pandemic (2017–2019). Only 16 cases of TIN were reported, of which 12 (75%) were male and 3 (18.8%) were female, and sex was unknown for one (6.3%) case. The age distribution showed that 10 (62.5%) cases were reported in the 61–90 years age group, followed by 4 (25.0%) in the 41–60 years group. Regarding the occupation of reporters, 7 (43.8%) were other healthcare professionals and 5 (31.3%) were physicians. Of the 16 TIN cases reported in this period, the outcome was hospitalization in 8 cases, while 1 outcome was life threatening (Figure 1).

**Table 2:** Demographic characteristics of TIN cases associated with statin use (2017–2019 vs. 2020–2024).

**Variable**	**Category**	**2017–2019 (*n* %)**	**2020–2024 (*n* %)**
Sex	Female	3 (18.8)	57 (54.8)
	Male	12 (75.0)	32 (30.8)
	Unknown	1 (6.3)	15 (14.4)
Age group	0–17	0 (0.0)	1 (1.0)
	18–40	0 (0.0)	9 (8.7)
	41–60	4 (25.0)	6 (5.8)
	61–90	10 (62.5)	72 (69.2)
	≥90	1 (6.3)	1 (1.0)
Reporter occupation	Unknown	2 (12.5)	2 (1.9)
	Physician (MD)	5 (31.3)	41 (39.4)
	Other Healthcare professionals	7 (43.8)	45 (43.3)
	Pharmacist	2 (12.5)	16 (15.4)

Reports of TIN cases associated with statin use between 2020 and 2024 are shown in Table 2. One hundred and four TIN cases were reported, 57 (54.8%) were female, 32 (30.8%) were male, and sex was unspecified in 15 cases (14.4%). The majority of affected individuals were aged ≥60 years, with 72 (69.2%) cases falling within the 61–90 age group. Only one pediatric case (age <18) and one case in a patient aged ≥90 were reported. Reporter occupation was most frequently listed as health professional in 45 (43.3%) cases, followed by physicians, 41 (39.4%) cases, and by 16 (15.4%) pharmacists. Of 104 TIN events, 68 (65.4%) were associated with hospitalization in 2020–2024. Five cases were deemed life-threatening, and one case resulted in death (Figure 1).

### Geographic distribution

**Figure 2:**
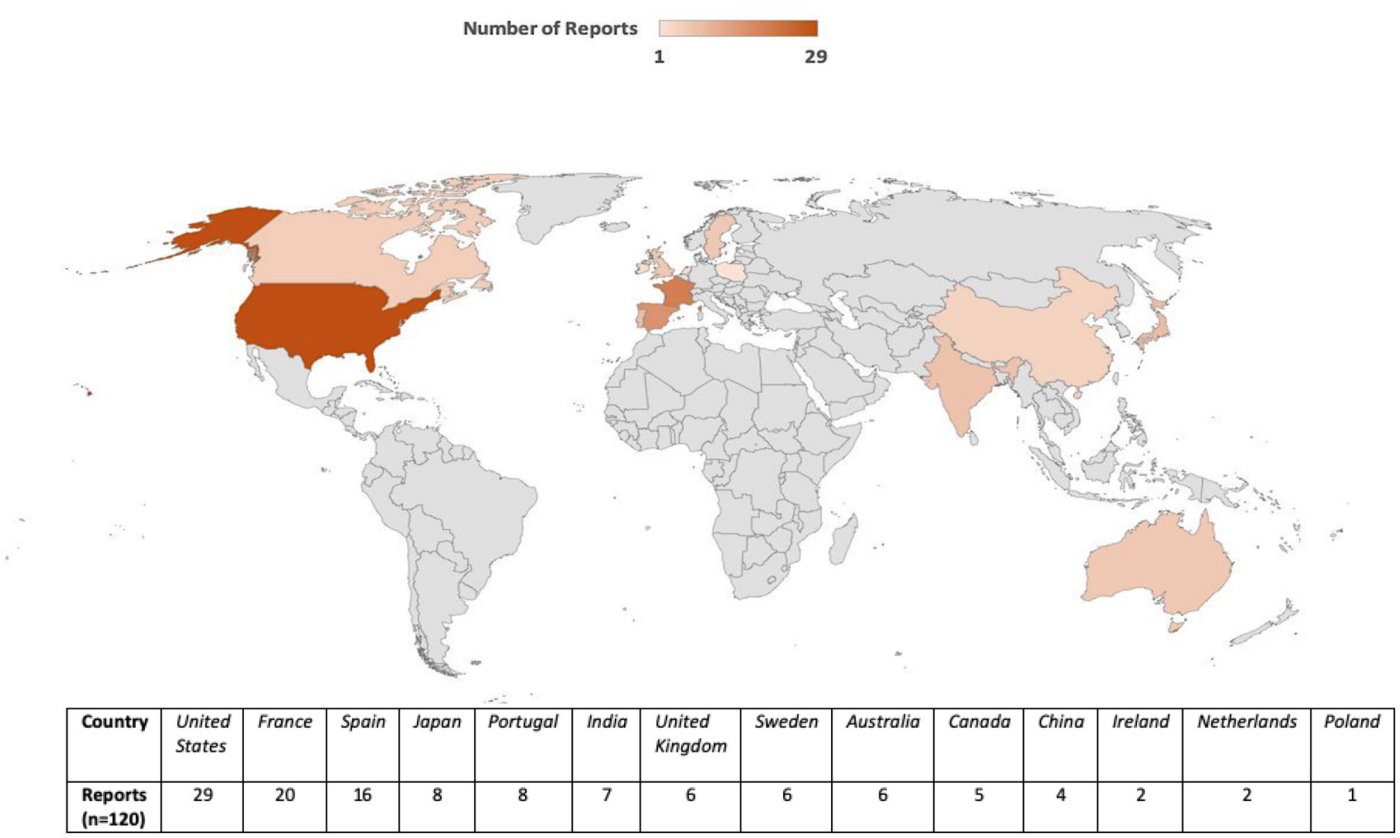
Global density distribution of TIN cases associated with statin use (2017–2024).

**Figure 3:**
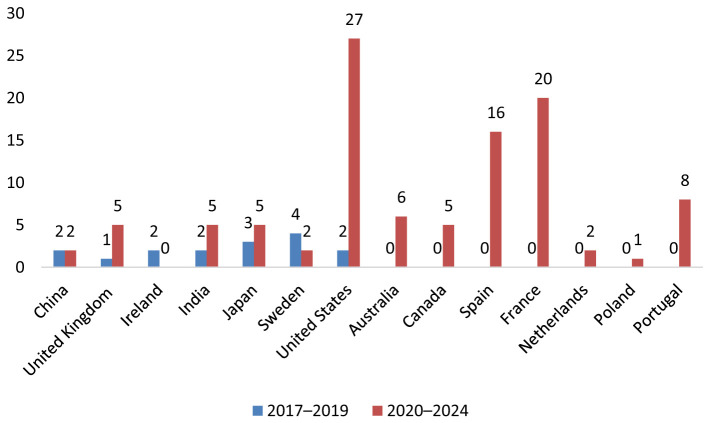
Geographic distribution of TIN cases associated with statin use (2017–2019 vs. 2020–2024).

Reports originated from 13 countries and the United States accounted for the largest proportion of cases (*n* = 29), followed by France (*n* = 20), Spain (*n* = 16), and Australia (*n* = 6) (Figure 2). This geographic variability may reflect differences in statin prescribing patterns, pharmacovigilance awareness, or reporting practices. The period-wise comparison of geographic distribution is depicted in Figure 3.

### Disproportionality analyses and adverse event reporting trends (2017–2024)

Table 3 displays the signal detection metrics for TIN associated with statin use for the complete study period (2017–2024). Based on the stratification before and after the COVID-19 pandemic, lesser TIN cases were reported with five cases in 2017, nine in 2018, and only 2 in 2019. Additionally, disproportionality analyses of the reports for the period before the COVID-19 pandemic showed that the ROR decreased from 2017 (0.80) to 2019 (0.07) and these ROR values were corresponding with similar values for PRR for these years. Moreover, no signal was shown by the EBGM and IC in the pre-COVID-19 period (e.g., IC value of −3.55 in 2019).

**Table 3:** Annual signal detection metrics for TIN associated with statin use (2017–2024).

**Year**	**TIN cases (*n*)**	**Reported ADEs with statins**	**ROR (95% CI)**	**PRR**	**EBGM**	**IC**
2017	5	7,646	0.80 (0.33–1.92)	0.8	0.81	−0.30
2018	9	9,639	0.41 (0.21–0.80)	0.41	0.43	−1.22
2019	2	12,089	0.07 (0.017–0.27)	0.07	0.08	−3.55
2020	12	10,088	1.13 (0.64–2.00)	1.13	1.13	0.17
2021	17	7,656	2.08 (1.29–3.35)	2.08	2.01	1.00
2022	16	6,976	2.89 (1.76–4.74)	2.89	2.71	1.44
2023	17	7,754	2.40 (1.49–3.88)	2.4	2.3	1.20
2024	42	8,503	5.77 (4.24–7.85)	5.75	5.32	2.41

In contrast, the period following the pandemic showed a significant increase in both the frequency and strength of signal detection metrics (Table 3). Firstly, the number of cases increased from 12 in 2020 to 42 by 2024. From 2021 to 2024, there was a significant increase in the ROR values (exceeding the standard threshold of 2.0) which suggests a potential safety signal. The ROR was 2.08 in 2021 (95% CI: 1.29–3.35), rose to 2.89 in 2022, then to 2.40 in 2023, and reached a peak of 5.77 in 2024 (95% CI: 4.24–7.85). Similar upward trends were seen in the PRR, which increased from 2.08 in 2021 to 5.75 in 2024. In addition, the EBGM, increased from 2.01 to 5.32, and the IC values rose from 1.00 to 2.41, thereby indicating a consistent and strong signal elevation for TIN associated with statin use.

## Discussion

Disproportionate analysis of FAERS data from 2017 to 2024 revealed a potential signal of TIN associated with use of statins in the era during and after COVID-19. Data was categorized as before the COVID-19 pandemic (2017–2019) and during and after the COVID-19 pandemic. The signal for TIN associated with statin use was not detected before the COVID-19 pandemic; however, it became evident from 2021 onwards as demonstrated by a consistent rise in the ROR, PRR, EBGM, and IC values with all values peaking in 2024. Although, causal relationship cannot be established, the observed signal and trend warrant increased clinical awareness and further investigation of this emerging safety concern.

The observed trend for TIN associated with statin use was significant and showed a strong signal for the period during and after the COVID-19 pandemic (2020–2024). The ROR values exceeded the conventional threshold of >2.0 from 2021 and reached 5.77 (95% CI: 4.24–7.85) in 2024. Moreover, the PRR, EBGM and IC values also rose correspondingly for the time period during and after the COVID-19 pandemic indicating a strengthening signal of association between TIN and use of statins. COVID-19 has been linked to broad lipidomic remodeling, including shifts in phospholipids such as phosphatidylethanolamines, which could influence immune activation and tubular stress responses (26). Statins can also modulate membrane organization and lipid-raft behavior, potentially altering host–drug interactions at the cell surface (27, 28). In parallel, pandemic-era dynamics—including changes in spontaneous-reporting behavior—may have influenced FAERS reporting patterns (29). Together, these factors could plausibly contribute to the stronger post-COVID-19 signal we observed; these hypotheses warrant mechanistic and pharmacoepidemiologic evaluation and should not be taken as evidence of causality. These findings are in alignment with a growing number of case reports that raised concerns about TIN with the use of statins. For instance, Londrino et al. reported a biopsy-proven case of rosuvastatin-induced TIN that resolved upon drug discontinuation and corticosteroid therapy (16). Another case from Saudi Arabia described by Panchangam also involved atorvastatin in TIN with patient developing concurrent rhabdomyolysis (14). Similarly, Annigeri and Mani documented two episodes of biopsy-confirmed TIN, first with atorvastatin and later upon re-challenge with rosuvastatin, suggesting a possible class effect (30). This association is further supported by a recent case report in which a 72-year-old female developed biopsy-confirmed TIN shortly after initiating rosuvastatin therapy; where the renal function improved significantly after discontinuation of statin and corticosteroid treatment, thereby reinforcing a likely immune-allergic mechanism (13). Moreover, rosuvastatin and atorvastatin were also among the most frequently reported drugs that cause TIN in a retrospective study that used data from Japanese Adverse Drug Event Report database (17). Furthermore, in 2024 the European Medicines Agency published the recommendations of the Pharmacovigilance Risk Assessment Committee (PRAC) requesting additional data from the marketing authorization holder regarding potential signal of TIN associated with use of rosuvastatin (19), thus supporting the credibility of our findings.

An important and concerning finding is the age distribution of TIN reports in the FAERS database for our study period. Most cases occurred in patients aged 61–90 years which is consistent with the typical age group for prescribing statin therapy for primary and secondary prevention of cardiovascular disease (18). Additionally, a significant difference in sex distribution of TIN reports was also seen in the period of 2020–2024 with most cases were seen among females as compared to 2017–2019 where male predominance was seen. While the reasons for this change in sex distribution is unclear, it may be possibly due to changes in prescribing trends, hormonal differences, body composition, or genetic predisposition which could contribute to sex-specific differences in drug metabolism and immune-mediated toxicity.

Geographically, the majority of reports were submitted from the United States, followed by France and Spain. Differences in prescribing patterns, national pharmacovigilance systems, and healthcare utilization likely contribute to this distribution. Additionally, variations in statin types available, public health surveillance sensitivity, and population demographics may further influence the global signal landscape. The significant proportion of cases from multiple continents adds to the external validity of the findings.

Moreover, the severity of outcomes further underscores the clinical importance of this signal: over 65% of TIN cases required hospitalization, five were life-threatening, and one case was fatal. These data should be interpreted cautiously; they may indicate clinically important outcomes in some reported TIN cases, but the overall burden cannot be inferred from FAERS. Given the small numbers and the absence of exposure denominators, together with possible stimulated/notoriety reporting and differences in clinical channeling, molecule-level comparisons are not reliable and do not support causal inference. These observations are best considered at the class level and should be viewed as hypothesis-generating rather than confirmatory. The diagnosis of acute tubulointerstitial nephritis is often delayed due to nonspecific clinical presentation, which may lead to ongoing renal inflammation and interstitial fibrosis, and progression to chronic kidney disease in up to 60% of patients, particularly in elderly patients or those on concurrent nephrotoxic agents (31).

The biological plausibility of statin-induced TIN is supported by histopathological descriptions in previous reports. TIN is primarily driven by immune-mediated inflammation of the interstitium and tubules, often drug-induced, accounting for up to 75% of cases (18). In our study, the clinical seriousness of outcomes alongside rising statistical signals supports the hypothesis that statins may trigger an immune-allergic renal response in susceptible individuals. This is further supported by previous case reports, such as those by Londrino et al., Annigeri and Mani, and González Martínez et al. where renal dysfunction resolved after discontinuation of statins (13, 16, 30). Importantly, such responses are often delayed and can be masked by the insidious nature of TIN, which lacks pathognomonic symptoms.

Comparatively, while the renoprotective effects of statins has been emphasized in chronic kidney disease and proteinuria (32), our analysis highlights a paradoxical subset of renal complications, likely linked to immunological idiosyncrasy rather than direct toxicity. Additionally, in a propensity-matched cohort analysis over 8.4 years, statin users exhibited significantly higher odds of nephritis/nephrosis/renal sclerosis compared with non-users, highlighting a potential long-term renal safety concern associated with statin therapy (33). This dual nature, protective in some contexts yet harmful in others necessitates a nuanced understanding of statin pharmacodynamics in high-risk populations.

These findings mirror the trajectory seen in another recent FAERS study, which examined pancreatitis with new antidiabetic medications and found an upward signal trend post-2019 (21). The parallels in signal behavior across drug classes during this period suggest a possible post-pandemic increase in awareness, changes in prescribing behavior, or shifts in immune-mediated adverse event reporting. The heightened attention to pharmacovigilance systems during and after the pandemic may also have played a role in increasing report volumes and improving case detection.

The strength of this study lies in its comprehensive and systematic use of four disproportionality metrics over an 8-year period. The temporal stratification before and after the COVID-19 pandemic also provides additional insight into evolving signal dynamics. Moreover, the identification of this signal across multiple countries suggests it may be observable across diverse healthcare settings, although differences in reporting practices limit generalizability. Importantly, this study adds value by highlighting an under-recognized but potentially serious renal adverse event reported in association one of the most widely prescribed drug classes globally. Moreover, integrating real-world pharmacovigilance data with published case reports helps contextualize the signal and its clinical presentation.

Nevertheless, several limitations must be acknowledged. FAERS data are subject to underreporting and variable data quality (10). Causality cannot be confirmed, as spontaneous reports lack temporal granularity and control groups. Diagnostic confirmation of TIN was not possible in most cases, as FAERS relies on coded adverse event terms rather than biopsy-verified diagnoses. Furthermore, co-medications and comorbidities are inconsistently reported, limiting our ability to control for confounding variables. The absence of detailed longitudinal clinical data precludes assessment of latency periods and response to withdrawal. Additionally, reporting bias and the Weber effect—where newer drugs or newly identified adverse effects are more frequently reported—must be considered (34). Increased awareness from published case reports or regulatory communications can lead to temporal spikes in reporting, which may overestimate signal strength. Despite these concerns, the consistency of signal increase across multiple years and statistical methods suggests that the observed association is unlikely to be incidental.

## Conclusion

Our study provides timely evidence suggestive of a potential association between statin use and TIN. Given the widespread prescription of statins globally, even a rare adverse event may translate into a significant public health concern. Clinicians may consider TIN in the differential diagnosis when patients on statins present with unexplained renal dysfunction, especially if accompanied by systemic symptoms. Further studies are warranted to validate these findings using longitudinal clinical datasets and renal histopathology data. Prospective studies and mechanistic investigations will be important to clarify whether a causal relationship exists and to identify patient-specific risk factors, which may ultimately inform clinical decision-making and pharmacovigilance policies.

## Data Availability

Publicly available datasets were analyzed in this study. This data can be found here: https://open.fda.gov/data/faers/.
